# Metabolic profiling of laser microdissected vascular bundles of *Arabidopsis thaliana*

**DOI:** 10.1186/1746-4811-1-2

**Published:** 2005-08-18

**Authors:** Martina Schad, Rajsree Mungur, Oliver Fiehn, Julia Kehr

**Affiliations:** 1 Max-Planck-Institute of Molecular Plant Physiology, Department Lothar Willmitzer, 14424 Potsdam, Germany; 2 UC Davis, 4321 GBSF Building Health Sciences Drive, Davis (CA) 95616, USA

**Keywords:** laser microdissection, GC-TOF MS, tissue-specific analysis, metabolite profiling, vascular bundle

## Abstract

**Background:**

Laser microdissection is a useful tool for collecting tissue-specific samples or even single cells from animal and plant tissue sections. This technique has been successfully employed to study cell type-specific expression at the RNA, and more recently also at the protein level. However, metabolites were not amenable to analysis after laser microdissection, due to the procedures routinely applied for sample preparation. Using standard tissue fixation and embedding protocols to prepare histological sections, metabolites are either efficiently extracted by dehydrating solvents, or washed out by embedding agents.

**Results:**

In this study, we used cryosectioning as an alternative method that preserves sufficient cellular structure while minimizing metabolite loss by excluding any solute exchange steps. Using this pre-treatment procedure, *Arabidopsis thaliana *stem sections were prepared for laser microdissection of vascular bundles. Collected samples were subsequently analyzed by gas chromatography-time of flight mass spectrometry (GC-TOF MS) to obtain metabolite profiles. From 100 collected vascular bundles (~5,000 cells), 68 metabolites could be identified. More than half of the identified metabolites could be shown to be enriched or depleted in vascular bundles as compared to the surrounding tissues.

**Conclusion:**

This study uses the example of vascular bundles to demonstrate for the first time that it is possible to analyze a comprehensive set of metabolites from laser microdissected samples at a tissue-specific level, given that a suitable sample preparation procedure is used.

## Background

Unlike unicellular organisms, plants and animals have evolved as complex organisms that are defined by distributing special vital functions to spatially separated organs and tissues. The distinct functions of tissues and organs result from the integrated activity of individual cells. Current approaches mostly ignore this fact by analyzing samples that consist of a variety of different cell types and thus average and dilute the information obtained. Parameters that define the function and the physiological state of cells include gene and protein expression, but also the complement of low-molecular-weight compounds such as lipids, carbohydrates, vitamins or hormones that carry out much of the cell's business. Therefore, in addition to transcriptomic and proteomic studies, a comprehensive metabolite analysis with high spatial resolution is essential to fully characterize the state of a certain tissue.

To achieve this, analysis of small solutes in individual plant cells has so far been performed after extracting picoliter-sized samples with glass microcapillaries [1] and specialized techniques have been developed to access the ingredients of such small-scale samples [2-5]. However, these techniques remained labor-intensive and require specialized skills and training.

The recent development of methods that allow the collection of enough individual cells to process with standard analytical methods is a key step to integrate high spatial resolution analysis into routine laboratory work. Laser microdissection (LM) [6,7] is meanwhile well-established with animal and human samples and allows the investigation of RNAs and proteins from specific cell types [8,9]. Recently, also plants have been successfully used in such experiments and information about gene [10-12] as well as protein expression [13] was obtained.

Before any tissue can be microdissected, histological sections have to be prepared. To this end the tissue is normally fixed and subsequently either embedded in paraffin [14,15] or cryosectioned [15-17]. However, the fixation and dehydration steps included in these procedures lead to an inevitable loss of small cellular components. Therefore, the resulting tissue sections do not allow measurements of the cellular distribution of metabolites and other low-molecular-weight compounds.

The intention of the current study was to develop a tissue processing procedure that enables tissue-specific measurements of metabolites in laser microdissected vascular bundle samples from *A. thaliana *stems with well-established metabolic profiling techniques. A combination of metabolite profiles with the already established gene and protein expression analysis from microdissected tissue samples will allow a comprehensive description of organisms under different developmental, biotic or abiotic conditions at a tissue-specific level, a further important step towards integrative biology.

## Results and discussion

### Preparation and collection of cell type-specific samples

The most important stage in the analysis of specific tissues or even single cells is the isolation of the target tissue away from adjacent cells. Using microcapillaries is an established option to collect the contents from living plant cells [1,3,18]. This sampling technique results not only in very small sample sizes in the picoliter range, but is also limited mainly to surface exposed cells. In this study, we have used laser microdissection (LM) as a powerful alternative sampling method, which allows a contamination-free collection of large homogeneous cell populations also from cells located deep inside the tissue [19].

To date, LM has been widely used, mainly to study cell type-specific gene expression and less frequently for tissue-specific protein analysis in animal tissues [8,9]. Recently, this technique was introduced into plant sciences to allow cell type- specific gene expression [10-12] and protein [13] profiling. Laser microdissection coupled to laser pressure catapulting (LMPC) is a method that is applicable to plant material and allows the contact-free collection of specific cells [20]. To perform successful LMPC, dry histological sections with reasonable morphology and cell integrity are needed. While preserving good morphology is required to distinguish the different cell types, it is also crucial to retain the analytes at their *in vivo *localization. The latter point particularly needs to be considered for the analysis of small substances like metabolites, since it is self-evident that they can easily relocate or get lost during tissue preparation.

In principle, there are two methods for tissue treatment available that are appropriate for subsequent protein or RNA analysis. The first is paraffin-embedding [21-23], which involves numerous steps to fix and dehydrate the tissue, leading to a complete loss of low-molecular-weight compounds. The second option is cryosectioning, which is commonly used to prepare sections for molecular studies of animal tissue, but often downgrades the histological architecture. Plant tissue is particularly prone to cell morphology damage, because ice crystal formation is facilitated in water-rich tissue. To overcome this problem, plant material is usually infiltrated with cryoprotection agents prior to freezing [11,12]. But as with paraffin fixation, the use of any solvent in the tissue preparation procedure results in a loss of small substances and makes cell type-specific metabolite profiling unfeasible.

**Figure 1:**
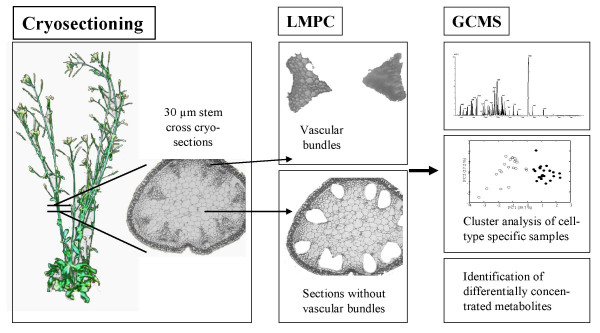
**Experimental strategy**.Outline of the strategy for tissue-specific metabolite profiling in *A. thaliana*. Stem cross cryosections were used for LMPC. Metabolites from microdissected vascular bundles and sections without vascular bundles, respectively, were extracted and analyzed by GCMS measurements.

For tissue-specific metabolite profiling of vascular bundles and sections without vascular bundles, we therefore followed the strategy illustrated in Figure 1. We employed a procedure where plant material is frozen without the addition of any protective compounds, and dried without chemical dehydration to avoid the loss or relocalization of small cellular components.

The morphology of the sections obtained from frozen *A. thaliana *stems using a cryostat was reasonable and completely sufficient to selectively excise vascular bundles by LMPC. However, getting decent morphology of stable tissue parts like vascular bundles is less complicated compared to other cell types, and it might also be more difficult to attain cryosections from more delicate plant organs like leaves or roots. Therefore cryopreservation and sectioning needs to be optimized depending on the plant species, organ and tissue type of interest.

**Figure 2:**
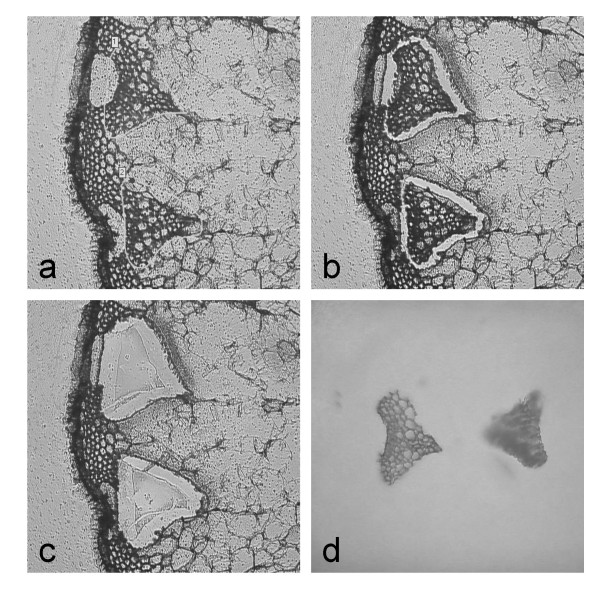
**Laser microdissection**.The process of laser microdissection coupled to laser pressure catapulting (LMPC) for collecting vascular bundles from 30 μm thick cross sections obtained after freezing and cryosectioning *A. thaliana *stems. (a) Vascular bundles are selected on the computer screen. (b) The laser beam cuts along the markings. (c) The cut cells of interest are catapulted off the slide by a defocused laser pulse and (d) are collected into a cap of a microfuge tube.

LMPC was performed to obtain tissue-specific samples as outlined in Figure 1. The stepwise process of sample collection from cryosectioned stem material is illustrated in Figure 2. First, vascular bundles were marked and thereby selected for excision by utilizing the P.A.L.M. specialized imaging software (Figure 2a). The cutting was then performed by a focused laser beam (Figure 2b), while the final tissue collection into the cap of a microfuge tube was achieved by a defocused laser pulse (Figure 2c and 2d). The lower limit of the tissue size that can be collected depends on the magnification and the minimal width of the laser beam which is in the range of 1 μm. In principle, LMPC can be used to isolate a few [19] and even single cells that are resolved e.g. in 40 × magnification (data not shown). The cap was filled with ethanol to inactivate metabolic enzymes during sample collection. This ethanol had to be refilled every 10 min due to evaporation of the solvent, since the collection of a sample consisting of 100 vascular bundles required approximately one hour. After collecting all vascular bundles from a section, the remnant sections without vascular bundles were scraped off into ethanol using a scalpel.

While there are studies demonstrating metabolite measurements in single individual or only a few plant cells, the resulting small sample volumes limited the subsequent metabolite analyses to a restricted number of metabolites that could be determined by enzymatic assays [1,24] or capillary electrophoresis [4,5]. These approaches allow the determination of sugars or amino acids with extreme sensitivity but require expertise that is only accessible in a few specialized labs. By employing GC-TOF MS, we intended to take advantage of a well-established analytical standard method that, although less sensitive than the methods described above and not suitable to measure metabolites in individual cells, has the advantage of allowing to comprehensively profile a variety of compounds from different classes simultaneously [25,26]. Moreover, GC MS-based platforms for comprehensive metabolite analysis are standard techniques accessible in many labs.

### Metabolite profiling

Dealing with such comparably small sample sizes as obtained by LMPC increases the danger of unwanted contaminations. Initial experiments using untreated plastic reaction tubes during all steps from tissue collection to derivatization led to GC-MS spectra from blanks that not only contained the expected artificial peaks resulting from derivatization and column bleeding, but also unexpected metabolite peaks as additional contaminants. The use of reaction tubes from different suppliers resulted in dozens of contaminant peaks in all method blank chromatograms. As a consequence, reaction tubes for all further experiments (0.5 ml plastic reaction tubes for LMPC as well as 0.2 ml glass vials for GC-MS derivatization) were washed twice with distilled water and dried. Pipette tips were rinsed once with ethanol directly before use. After sample collection, vortexing and centrifugation, supernatants were transferred to glass vials where the derivatization was carried out. These precautions drastically improved the quality of blanks and were essential for metabolite profiling of small sample amounts.

Next, the influence of the sample preparation procedure on the metabolite composition was investigated. To this end we compared samples that were (a) dissolved in ethanol directly after cryosectioning or (b) cut, transferred to slides, stored at 4°C, and used for LMPC. As expected, some differences in the metabolite profiles of the two sample types were observable: 11 of 72 metabolites identified in measurements from 5 complete stem cross sections (10 replicates) appeared to be sensitive to the sample preparation procedure. These changes were statistically significant (P < 0.05) and showed an at least 1.5 fold increase or decrease in LMPC samples as compared to cryosectioned control samples (Table 1). These observed changes are probably caused by biological and chemical processes during storage, processing and microdissection, since all these steps were carried out at room temperature without air exclusion. Therefore, samples have to run through identical procedures to allow comparisons of metabolite levels. Absolute quantities of certain metabolites have to be treated with caution. Dehydroascorbate (the oxidized form of vitamin C), for example, was found to be strongly diminished by the tissue preparation process (Table 1), putatively due to oxidation by air. In LMPC collected vascular bundles and sections without vascular bundles, dehydroascorbate was completely absent.

**Table 1:** List of metabolites influenced by the sample preparation procedure. Metabolite amounts in stem cross sections. To analyze the influence of tissue processing and laser microdissection on metabolite profiles, complete fresh cryosections and sections after drying and laser microdissection (consisting of vascular bundles and the remaining tissue) were compared. Metabolites with significant differences (*P *< 0.05) in cryosections and laser-microdissected sections (>1.5) are listed.

**Metabolite**	**CAS**	**Ratio of fresh cryosections vs. complete laser microdissected sections**
glycerol	56-81-5	0.6
urea	57-13-6	0.6
lauric acid	143-07-7	0.6
linoleic acid	60-33-3	1.6
succinic acid	110-15-6	1.7
myo-inositol	87-89-8	1.7
shikimic acid	138-59-0	2.0
ethanolamine	141-43-5	2.5
gamma-aminobutyric acid	56-12-2	2.5
glucose	50-99-7	3.9
dehydroascorbic acid	490-83-5	8.5

For tissue-specific GC-TOF MS measurements, we prepared stem cross sections of five *A. thaliana *plants and collected by LMPC five replicates of about 100 vascular bundles (~ 5,000 cells) from each plant into ethanol. Additionally, 10 of the remaining vascular bundle-depleted sections from each plant were scraped off the slides and also collected into ethanol. The supernatants were dried, derivatized with MSTFA and subjected to GC-TOF MS metabolite profiling. Due to the limited sample amounts available, derivatization of the metabolites was done in a volume of only 10 μl. For the same reason, the injection was carried out without sample wash steps.

### Data evaluation

**Figure 3:**
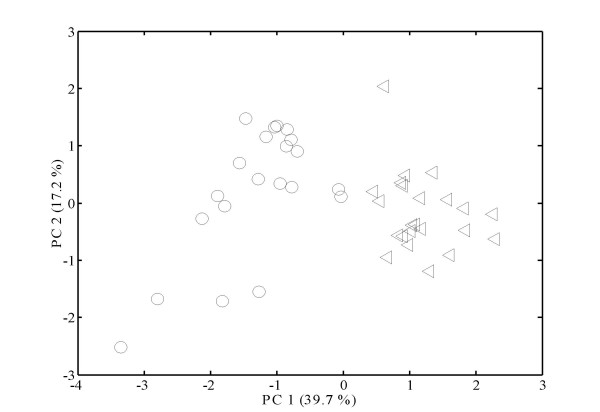
**Statistical evaluation of the metabolite data**.Principal Components Analysis (PCA) of metabolite samples generated from *A. thaliana *stem tissue. The PCA score plot for principal component 1 (PC1) versus principal component 2 (PC2) is presented. The circles represent microdissected vascular bundle tissues (21 samples) whilst the triangles are samples from sections without vascular bundles (23 samples).

Using the GC-TOF MS approach, 68 metabolites could be reliably identified in vascular bundles and 65 in sections without vascular bundles. This number of identified metabolites is reasonable considering the small number of cells (~5,000) used for the measurements. The obtained data sets were investigated in more detail to find out differences and similarities in the metabolic profiles from vascular bundles and the surrounding tissue. First, we carried out a principal component analysis (PCA) to see if logical grouping in the data set could be related to maximum variance. This unsupervised multivariate data analysis generates new variables, principal components (PC), that attempt to express the overall variance in the original data. When plotting the tissue-specific metabolite data, it was immediately obvious that vascular bundles and sections without vascular bundles were well separated and had their own distinct metabolite profiles (Figure 3), indicating that the localization of metabolites was retained during sample processing.

**Figure 4:**
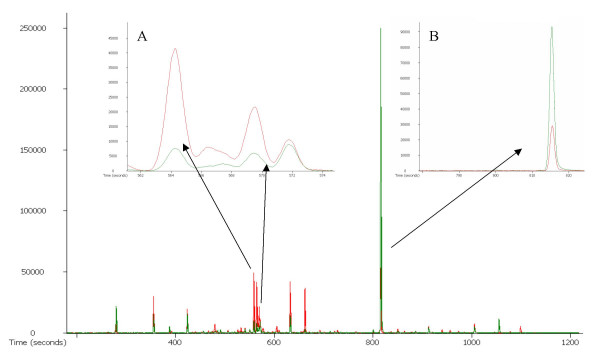
**Example chromatogram**.Entire chromatogram for m/z 217, an ion trace for sugars and sugar alcohols. The vascular bundle sample is shown in green and the sample without vascular bundle in red. (a) shows an example of depleted sugars in vascular bundles – fructose (retention time 564.19 s) and glucose (retention time 569.419 s) while (b) illustrates the zoomed-in sucrose peak (retention time 815.369 s) being enriched in vascular bundles.

Student t-test comparisons between the data sets revealed that about half of the identified metabolites were significantly different and either enriched or depleted in vascular bundles compared to sections without vascular bundles (Table 2), including six metabolites which were shown to be sensitive to the sample preparation procedure (Table 1). Figure 4 shows examples of chromatograms of vascular bundles and sections without vascular bundles for the mass to charge ratio (m/z) 217. This ion trace is used to look at sugars and sugar alcohols. The insets (Figures 4a and 4b) illustrate examples of metabolites being enriched and depleted, respectively, in vascular bundles. Seventeen metabolites were significantly depleted in vascular bundles, while 16 metabolites were enriched in vascular bundles and three metabolites, oxoglutarate, glyceraldehyde and glycerone, were even exclusively found in this tissue type. We are not aware of anything in the literature that would account for these metabolites being exclusively localized to vascular bundles.

**Table 2:** Metabolites differentially concentrated in vascular bundles and sections without vascular bundles. Metabolites that were differentially concentrated in vascular bundles and sections without vascular bundles (with *P *<0.05 and ratio <0.67 or >1.5) are listed. vb: found only in vascular bundles, ** metabolites which appear to be sensitive to the sample preparation procedure (see Table 1).

**Metabolite**	**CAS**	**Ratio of vascular bundles vs. sections without vascular bundles**
stigmasterol	83-48-7	0.1
gamma-aminobutyric acid**	56-12-2	0.3
ethanolamine**	141-43-5	0.3
galactose	59-23-4	0.4
fucose	634-74-2	0.4
linolenic acid	463-40-1	0.4
glucose**	50-99-7	0.4
fructose	57-48-7	0.4
phenylalanine	63-91-2	0.5
linoleic acid**	60-33-3	0.5
leucine	61-90-5	0.5
benzoic acid	65-85-0	0.6
urea**	57-13-6	0.6
isoleucine	73-32-5	0.6
valine	72-18-4	0.6
lignoceric acid	557-59-5	0.6
mannose	3458-28-4	0.7
heptadecanoic acid	506-12-7	1.6
aspartic acid	56-84-8	1.7
malic acid	97-67-6	1.7
adipic acid	124-04-9	1.8
proline	147-85-3	1.9
sucrose	57-50-1	1.9
lauric acid**	143-07-7	1.9
raffinose	512-69-6	2.1
isocitric acid	320-77-4	2.2
oleic acid	112-80-1	2.3
myristic acid	544-63-8	2.4
glycine	56-40-6	2.8
6-amino caproic acid	60-32-2	3.5
citric acid	77-92-9	3.7
trans-squalene	111-02-4	4.4
phosphate	14265-44-2	5.2
oxoglutarate	328-50-7	vb
glyceraldehyde	367-47-5	vb
glycerone	96-26-4	vb

Sugars accounted for one class among the metabolites with tissue dependent differences. While reducing sugars like glucose, fucose and fructose were depleted in vascular tissue, the non-reducing sugars sucrose and raffinose were enriched. This distribution of sugars is characteristic for phloem sap that probably accounts for a great portion of vascular bundle metabolites.

A further class of compounds that showed different levels in vascular bundles compared to the surrounding tissue was amino acids. From this group glutamate, aspartate and glutamine are the major amino acids distributed through the phloem tubes in *A. thaliana *[27] and therefore could be expected to be enriched in vascular bundles. In this study, we found only aspartate significantly enriched in vascular bundles compared to the surrounding tissue while glutamate and glutamine were indeed abundant in vascular bundles, but similar levels were also detected in sections without vascular bundles. These findings are unlikely to be caused by the sample treatment because none of these amino acids belonged to the group of substances influenced by the sample processing procedure (Table 1).

## Conclusion

This study demonstrates for the first time that laser microdissection can be successfully applied to analyze the spatial distribution of metabolites within plant tissues. The application of a suitable sample preparation protocol, omitting any solute exchange steps, followed by LMPC makes metabolite profiling of 100 vascular bundles, equivalent to only 5,000 cells, by standard GC-TOF MS measurements feasible. In principle this method should be applicable to a wide range of cells and tissues given that sufficiently good morphology is obtained following the introduced cryosectioning procedure. In combination with the previously described RNA and protein expression profiling, cell type-specific metabolite profiling will allow a comprehensive characterization of distinct tissues, an essential step towards a thorough understanding of gene functions.

## Methods

### Preparation of tissue sections and laser microdissection

*Arabidopsis thaliana *(Col-0) plants were grown on soil in a growth chamber under short day conditions (8 h light, 20°C and 16 h dark, 16°C, 75% RH) for four weeks and then in the greenhouse under long day conditions (16 h light, 20°C, 60% RH and 8 h dark, 18°C, 75 % RH). Stems of 6 week-old plants were frozen in liquid nitrogen and transferred to a cryostat (Microm, Waldorf, Germany) cooled to -30°C. Using a scalpel, 15 mm-long stem pieces were cut. Stem pieces were glued onto the sample plate by employing Neg-50 (Richard-Allan Scientific, Kalamazoo, MI, USA), a water-soluble frozen section medium. Using the cryostat, 30 μm sections were cut and transferred to glass slides where they dried within seconds at room temperature.

For microdissection, the P.A.L.M. Laser Microbeam System (Bernried, Germany) was employed. This system consists of a low heat UV (337 nm nitrogen) laser and an inverted microscope. Cells were selected using the graphics tools of the P.A.L.M. RoboSoftware. After selection, vascular bundles were isolated by the laser microbeam and afterwards collected by laser pressure catapulting into the lid of a 0.5 ml reaction tube (Eppendorf, Hamburg, Germany) filled with 50 μl ethanol (Merck, Darmstadt, Germany), placed in a holder closely above the slide. After collection of vascular bundles, the remaining sections were scraped off the slides using a scalpel into ethanol (subsequently called sections without vascular bundles). The cell numbers were estimated from the number of vascular bundles collected and the cell number counted in one representative vascular bundle.

Both types of samples were vortexed and centrifuged for 5 min at 14.000 rpm. Supernatants were collected in 0.2 ml glass vials (Chromacol, Herts, United Kingdom) and vacuum dried. All used reaction tubes, including their lids, were washed twice with distilled water and dried before use. More details of quality control procedures during sample preparation are provided in the text main body. Five parallel plants were employed. From each plant, five replicates of 100 vascular bundles and 5 sections without vascular bundles were used for metabolite analysis. As negative controls, ethanol without microdissected material was processed in parallel to the samples.

### Metabolite profiling

For GC-TOF MS (Leco Pegasus II GC-TOF mass spectrometer; Leco, St. Joseph, MI, USA) analysis, the vacuum dried samples were dissolved in 1 μl methoxamine hydrochloride (20 mg/ml pyridine) and incubated at 30°C for 90 min with continuous shaking. Then 9 μl of N-methyl-N-trimethylsilyltrifluoroacetamid (MSTFA) were added to derivatize polar functional groups at 37°C for 30 min. The derivatized samples were stored at room temperature for 120 min before injection. GC-TOF MS analysis was performed on an HP 5890 gas chromatograph with tapered, deactivated split/splitless liners containing glass wool (Agilent, Böblingen, Germany) and 1.5 μl splitless injection at 230°C injector temperature. Before each injection, the liner was rinsed with a pure MSTFA injection (1 μl). Sample injection was carried out without sample wash steps due to the limited amount of total sample volume. The GC was operated at constant flow of 2 ml/min helium and a 30 m 0.32 mm id 0.25 μm MDN35 column (Macherey-Nagel, Düren, Germany). The temperature gradient started at 80°C, was held isocratic for 2 min, and subsequently ramped at 15°C /min to a final temperature of 330°C which was held for 6 min. Twenty spectra per second were recorded between m/z 85–500. Peak identification and quantification were performed using the Pegasus software package ChromaTOF 1.61 (Leco). A reference chromatogram was defined that had a maximum of detected peaks over a signal/noise threshold of 5 and used for automated peak identification based on mass spectral comparison to a standard NIST 98 library and own customized mass spectral libraries. Automated assignments of unique fragment ions for each individual metabolite were taken as default as quantifiers, and manually corrected where necessary. All known artifact peaks caused by column bleeding or phtalates and polysiloxanes derived from MSTFA hydrolysis were manually identified and removed from the results table. Remaining metabolite data were normalized to the total area of all detected metabolites and log-transformed. Due to the utmost requirements of low sample volumes and chemical background, no further internal standards were added. Statistical analyses were performed using Matlab version 6.5 (The MathWorks, MA, USA).

## Competing interests

The author(s) declare that they have no competing interests.

## Authors' contributions

MS carried out the optimization of sample preparation for GC-TOF MS measurements, collected samples by LMPC, participated in data evaluation and drafted the manuscript. RM participated in LMPC sample collection, GC-TOF MS measurements and data analysis. OF participated in GC-TOF MS optimization, data evaluation, and manuscript drafting. JK conceived of the study, arranged its design and coordination and was involved in manuscript writing. All authors read and approved the final manuscript.
